# Impact of Bedding Volume on Cage Microclimate and Behavior in 129SV and Desmin-Knockout (Des−/−) Mice

**DOI:** 10.3390/ani16111585

**Published:** 2026-05-23

**Authors:** Evdokia Minoudi, Efthymios Paronis, Konstantinos Konstantinidis, Nikolaos Sarikavazis, Pavlos Alexakos, Dimitrios Chaniotis, Ioanna Kostavasili, Nikolaos Kostomitsopoulos, Chrysa Voyiatzaki

**Affiliations:** 1 Laboratory Animal Unit, Biomedical Research Foundation of the Academy of Athens, 11527 Athens, Greecenkostom@bioacademy.gr (N.K.); 2 Laboratory of Molecular Microbiology and Immunology, Department of Biomedical Sciences, University of West Attica, 12243 Egaleo, Greece; 3 Laboratory of Biology, Department of Medicine, Democritus University of Thrace, 69100 Alexandroupolis, Greece; 4 Laboratory of Anatomy-Pathological Anatomy & Physiology-Nutrition, Department of Biomedical Sciences, University of West Attica, 12243 Egaleo, Greece

**Keywords:** mice, cage microenvironment, environmental enrichment, bedding, temperature, humidity, intra-cage behavior, digging, bedding soiling

## Abstract

Standard laboratory housing often restricts the expression of natural behaviors in rodents, raising concerns regarding animal welfare and the translational value of experimental data. The current study investigated whether increasing cage bedding volume functions as an effective, low-impact environmental enrichment strategy that enhances welfare without confounding physiological parameters. An in-depth analysis was conducted comparing the effect of Deep Bedding (DB; 1600 mL) versus Normal Bedding (NB; 400 mL) on the microenvironment, physiology, and behavior of wild-type (129SV) and desmin-knockout (Des−/−) mice. Results demonstrated that deep bedding acts as a significant environmental buffer, effectively decoupling intra-cage temperature and humidity from ambient room fluctuations. While physiological parameters, including body surface temperature, body weight, and food intake, remained stable across bedding conditions, behavioral analysis revealed a robust upregulation of species-specific fossorial activities, such as digging and burrowing, in the DB group. This suggests a beneficial “bioenergetic reallocation” where energy is directed toward natural behaviors rather than thermal stress responses. Furthermore, deep bedding significantly improved cage hygiene through the mechanical sequestration of waste. These findings indicate that deep bedding serves as a multifactorial refinement strategy that supports animal welfare and hygiene without increasing experimental variability, proving safe even for physiologically sensitive models like Des−/− mice.

## Simple Summary

The current study addresses a central challenge in laboratory animal research: conventional housing conditions for mice are often restrictive, limiting natural behaviors and potentially compromising both animal welfare and the reliability of scientific findings. The aim was to evaluate whether increasing bedding volume, a simple and practical modification, could enhance living conditions without adversely affecting key experimental outcomes. Mice housed in standard versus deep bedding were compared across environmental, physiological, and behavioral measures, including cage temperature and humidity, body weight, food intake, hygiene, and activity patterns. The results demonstrate that deep bedding creates a more stable and buffered cage microenvironment, reduces surface waste through improved absorption and burial, and promotes natural behaviors such as digging and burrowing. Importantly, these benefits were achieved without meaningful changes in body temperature, food intake, or most physiological parameters, indicating minimal impact on experimental variability. The study concludes that deep bedding represents an effective, low-impact refinement that improves animal welfare while preserving data integrity, even in sensitive disease models. These findings are valuable to society as they support more ethical animal research practices and enhance the reliability of biomedical data, ultimately contributing to better translation of research outcomes into advances in human health.

## 1. Introduction

It is well established that the restrictive nature of housing systems used for laboratory rodents limits their ability to express natural behaviors [1,2,3]. Environmental enrichment involves modifying housing conditions in various ways to optimize animal welfare and health [4]. The NIH Office of Animal Care and Use, while reporting Guidelines for General Species Environmental Enrichment (2023), defines enrichment as the “increase in the complexity of the environment in which the animal lives, with the goal of enhancing the animal’s welfare”. Numerous methods to achieve environmental enrichment have been proposed, each serving different purposes. For instance, enrichment objects and toys enhance memory and learning [5]. In a more recent metareview of 737 relative articles, authors concluded that the positive effects and mechanisms of environmental enrichment in dementia rodent models were noticeable [6]. Social housing minimizes stress and boredom and creates active engagement and it is highly anticipated by mice, while nesting materials, shelters and nest boxes reinforce the performance of natural behaviors, such as nesting and gnawing [7]. Enrichment has been associated with a positive outcome for animals with neurodegenerative diseases such as Huntington’s and Alzheimer’s, as well as stroke and brain damage recovery [8]. Such effects of enrichment on animals with neurodegenerative and neurological disorders have been reported recently [9]. Therefore, enrichment, especially when properly designed, has the purpose of effectively improving animal welfare, in a variety of ways [8].

It is important to note that because enrichment provides physical, cognitive, and sensory stimulation [10], animals do not respond to it uniformly. Factors such as sex, age, and phenotype can play a determining role in shaping these responses, resulting in variable behavioral and neurobiological outcomes. As a result, concerns are frequently raised about the potential for increased data variability in experiments that incorporate enrichment methods [11,12,13]. Conversely, unenriched housing conditions fail to adequately support animal welfare, which is essential for high-quality translational research [14]. Moreover, the use of laboratory animals that experience various forms of stress due to conventional housing systems raises questions about how well these models can represent humans, who do not experience comparable stressors [15]. While the use of enrichment toys and objects has been correlated with overall promotion of rodent welfare, practical limitations—such as lack of space in the cage—should be considered, and the use of enrichment practices that resemble aspects of a species’ natural environment may be preferable [16]. Taken together, these considerations highlight the need for minimally stimulating, welfare-supportive, low-impact enrichment strategies that support laboratory rodent welfare while preserving the integrity of experimental data.

Mice respond strongly to modifications of their environment, and the thickness of bedding is one factor that can greatly improve their welfare. Evidence support that greater bedding volumes can be attributed to the expression of natural behaviors such as digging [17], improvement in thermoregulation opposed to shallow bedding [18], and the possibility of continuous microenvironment alteration, which is crucial elements of animal welfare. Even minor environmental modifications, such as bedding type, can influence rodent sleep behavior and substrate-related preferences, with reported effects on sleep architecture including slow-wave sleep (SWS) in rats [19]. Indeed, Freyman et al. reported that C57Bl/6 and BALB/c mice have shown preference in deep bedding in comparison to shallow bedding environments, as they have spent significantly more time in cages with more bedding material during the preference test [20]. Especially during the light phase, when mice tend to rest, preference for deep bedding was increased, suggesting that more bedding material creates a more comfortable environment for mice to sleep [20]. Both female and male mice showed preference to deep bedding, revealing no sex differences, even though a different degree of preference was observed between the two strains [20]. Additionally, they found that ammonia concentrations declined as bedding depth increased, in fact, seven days after a cage change, ammonia was completely absent in cages containing the deepest bedding [20,21]. Mice housed on the deepest bedding also showed higher body temperatures and reduced food consumption, while females of both strains exhibited improved feed conversion efficiency and lower corticosterone levels [20,21]. Deep bedding can be perceived as a naturalistic enrichment method, interesting for testing on mice that are more sensitive to environmental stimuli.

The current article investigates whether an increase in the cage bedding volume could potentially work as an effective enrichment method that enhances animal welfare without strongly altering experimental parameters. Different strains of mice react differently in such environmental modifications. Digging and burrowing are considered motivated, species-typical behaviors in mice, especially in strains with hole-nesting ancestry (e.g., C57BL/6 mice). While strains such as BALB/c mice exhibit more surface-nesting behavior, the phylogenetic relationship of 129SV-derived lines to these categories remains unclear. 129SV mice generally show reduced activity and increased anxiety-like behavior [22], and so does the Des−/− model, derived from a 129SV background, share this limitation. The genetically altered, knock-out Des−/− mouse that lacks desmin is an interesting mouse model, used in cardiovascular diseases and skeletal myopathies. Even though Des−/− mice grow normally and are fertile, cardiomyopathy occurs after birth resulting in low tolerance in exercise and life span [23]. Given that even modest changes in ambient temperature can substantially influence cardiovascular parameters such as blood pressure, heart rate, pulse pressure, and heart rate variability [24,25,26], it is relevant to evaluate how increasing bedding volume might affect the physiology and welfare of this sensitive mouse model.

Within this framework, the present study aims to examine how increasing bedding depth influences the cage microenvironment and to assess whether this approach can serve as an effective, non-stimulating form of environmental enrichment that enhances housing conditions and overall welfare in laboratory mice. Moreover, we hypothesize that deep bedding may provide particular benefits for desmin-knock-out (Des−/−) mice, offering a strain-specific enrichment strategy, while also improving the living conditions of wild-type 129SV mice. By evaluating these effects, this work seeks to establish deep bedding as a practical and welfare-oriented refinement in laboratory rodent care.

## 2. Materials and Methods

### 2.1. Animals

A total of 64 mice—comprising 32 wild-type (129SV) and 32 desmin-null (Des−/−) mice of both sexes—were utilized in this study. At the onset of the experiment, the individuals were 10–12 weeks of age with a mean body weight of 23.06 ± 1.8 g. To systematically assess the microenvironmental variables, the animals were allocated into 16 experimental cages at a standardized density of 4 mice per cage, stratified evenly by genotype, sex, and assigned bedding depth. Furthermore, to ensure behavioral and physiological baseline stability, all animals underwent a 14-day acclimatization period within these specific housing conditions prior to the initiation of data collection.

### 2.2. Housing Conditions

All experiments were performed in the Biomedical Research Foundation of the Academy of Athens (BRFAA). The facility is registered as a “breeding” and “experimental” facility (Reg. Numbers: EL BIO 01 and EL 25 BIO 03, respectively) according to the Greek Presidential Decree 56/2013, which harmonizes National Legislation with the European Directive 2010/63 on the protection of animals used for scientific purposes. All applicable National guidelines for the care and use of animals were followed. Cages were kept in the same animal room with air supply, 15 Air Changes per Hour (ACH), an ambient room light intensity of 300 lux measured one meter above the floor in the middle of the room, and a color temperature of 4100 K, as well as positive air pressure of 0.6 Pa within the room. To account for the relatively high ambient light, light intensity was measured both outside and inside the cages prior to the experiment using a calibrated lux meter (LX1010BS). Because the animals were housed in tinted polysulfone cages, the intra-cage light intensity was reduced by approximately two-thirds compared to the external environment, resulting in a suitably dimmed exposure of 70–80 lux for the mice. Room conditions were continuously monitored through the central Building Management System (BMS) of the animal facility.

Animals were bred and maintained in a specific pathogen-free, temperature- and humidity-regulated room (21 ± 2 °C; 55% ± 10%), and a 12/12 h light/dark cycle with lights off at 19:00 h and no twilight period. Mice were housed in individually filter top cages (1284 L, H-Temp™ Polysulfone—PSU, Techniplast, Varese, Italy), at a stocking density of 4 mice per cage [caging dimensions (L × W × H): 365 × 207 × 140 mm, floor area = 530 cm^2^]. The stocking density was decided based on data of a relative study, examining the effectiveness of nesting material [27].

To accurately evaluate deep bedding as the sole environmental enrichment variable, no additional enrichments—such as nesting material, gnaw blocks, or nest boxes—were provided. This baseline setup was intentionally chosen to isolate the specific effects of bedding depth under highly standardized conditions, avoiding the unwanted experimental variability often introduced by the uneven use and maintenance of supplemental enrichments. This standardized baseline housing met the minimum regulatory requirements stipulated by National guidelines and European Directives for laboratory animal care.

All mice had *ad libitum* access to filtered tap water in drinking bottles and a vacuum-packed pelleted rodent chow that contained 18.5% protein, 5.5% fat, 4.5% fiber, and 6% ash (4RF22, Mucedola, Milan, Italy). The bedding in each cage consisted of autoclaved corncob bedding (Rehofix MK 2000, J. Rettenmaier & So, Rosenberg, Germany). The cages were cleaned and autoclaved once every two weeks.

### 2.3. Experimental Design

Animals were housed in two experimental housing conditions:

Non enriched conditions: cages contain 400 mL (200 g, 3 cm in depth) of bedding material (normal bedding, NB). Given the cage floor area of 530 cm^2^, this corresponds to a bedding density of 0.38 g/cm^2^. This volume was chosen as it represents a common conventional baseline aimed at sufficiently covering the cage floor.

Enriched conditions: cages contain 1600 mL (800 g, 12 cm in depth) of bedding material (deep bedding, DB), corresponding to a bedding density of 1.51 g/cm^2^.

**Figure 1:**
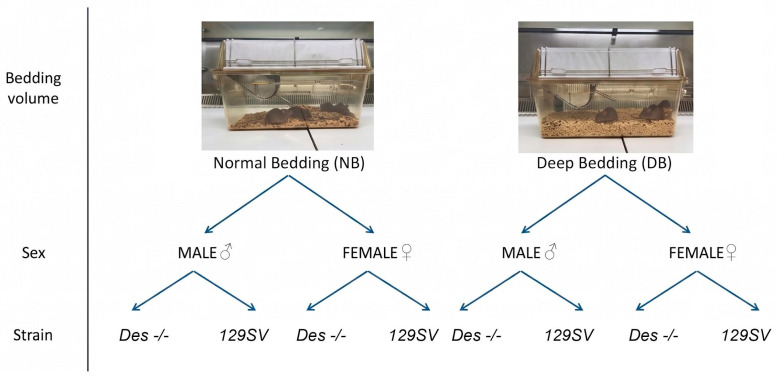
Diagram illustrating the allocation of mice in eight groups, based on three parameters (bedding volume, sex, strain).

Wild-type 129SV and desmin-knockout Des−/− (129SVNULL-MPLF24, 129SVNULL-MPL27) mice, of both sexes were housed in the two different housing conditions, allocating mice in 8 groups (2 genotypes × 2 sexes × 2 housing conditions) (Figure 1). To ensure compliance with ARRIVE 2.0 guidelines [28], animals were randomly allocated to these experimental groups to ensure a balanced representation of sex, strain, and baseline body weight. Experimental unit is the cage, as environmental enrichment is a condition applied to the whole cage and 4 mice were allocated per cage. The sample size (total number of experimental units) was 16 cages, 2 cages or 8 mice per group. Animals were monitored daily by two independent observers. To minimize potential environmental confounding, static cages were periodically repositioned within the room after every bedding change (every 14 days) according to a randomized scheme. This counterbalancing strategy was employed to reduce positional effects such as light exposure, airflow, or proximity to door openings. Intra-cage temperature and humidity were instantaneously measured daily, using cage thermometers (Life Flexy Indoor Thermometer & Hygrometer Tabletop, FLEXY) that were placed on the internal surface of the lid, over the food pellets.

Body weight and food intake were measured biweekly, immediately following behavioral observations and prior to cage cleaning. Consequently, each animal contributed one measurement per two-week period, which were the values utilized for downstream analyses. Food intake was assessed at the cage level. Specifically, a known quantity of food (900 g) was provided to each cage at the beginning of the two-week period. At the time of cage changing, the remaining food was weighed, and the difference from the initial amount was calculated to determine the total food consumption per cage. This total value was then divided by the number of animals per cage (*n* = 4) to yield an estimated individual food intake. Although the NB volume represents a minimal substrate layer for a biweekly cage-change schedule, daily health monitoring by animal care staff ensured that cage conditions did not exceed acceptable hygienic thresholds requiring premature intervention.

### 2.4. Body Surface Temperature Measurement

Body surface temperature was measured at 13:00 (in the middle of the light period) before the cage changing (every 14 days), as the changing process itself is a known stressor that acutely increases body temperature. To minimize stress prior to these measurements, mice were housed in static cages, thereby avoiding the noise and mechanical disturbance typically associated with moving ventilated IVC cages during un-racking. Furthermore, prior to measurement, cages were carefully opened and the animals were left undisturbed for a short acclimation period, allowing any acute stress levels to subside. In total, 192 measurements of body surface temperature were taken from mouse individuals during this study, which were sequentially averaged per cage experimental unit. The use of a digital non-contact infrared thermometer (NC100, MICROLIFE, Vilnius, Lithuania) was preferred to measure body surface temperature, because it is a non-invasive, low-stress, and inexpensive method. The thermometer was directed at the sternum of mice to minimize the deviation of body surface temperature from core body temperature [29,30]. During the actual measurements, handling of mice took no longer than a few seconds, as it has been shown that long durations of handling increase temperature in a stress-relative way [31,32].

### 2.5. Assessment of Level of Bedding Surface Soiling

Images of the upper surface of the bedding material in every group were taken before every biweekly cage change. Images were then analyzed with the use of Image Pro (Image Pro Plus version 4.1; Media Cybernetics; Rockville, MD, USA) as a method to quantify the level of bedding surface soiling, in order to locate any potential difference as a result of the implementation of the current enrichment method. Soiling was identified through the color difference between soiling and bedding material. Appropriate modifications in image contrast were made, for better evaluation. Fecal soiling was assessed by measuring the area of fecal deposits relative to the total outer bedding surface in square pixels. These values were then converted to percentages to facilitate comparison between groups.

### 2.6. Intra-Cage Behavior

Behavioral assessment of mice inside the home cage was utilized to evaluate animal interaction with the bedding microenvironment, utilizing an *a priori* ethogram adapted from standard rodent behavioral frameworks [20,33] (Table 1). This ethogram focused on core behavioral categories relevant to the aims of the study, including activity, maintenance, social interactions, and species-specific behaviors. Abnormal repetitive behaviors (e.g., stereotypies) and specific states such as ‘inactive but awake’ (IBA) were not explicitly included as separate categories; instead, general inactivity was captured under the broad ‘resting’ category without distinguishing between sleep and quiet wakefulness.

Behavioral observations were conducted daily starting at 13:00 by two independent observers. Each cage (housing four mice) was observed for a continuous 10 min period. During this time, all four animals within the cage were monitored simultaneously using a group observation approach, rather than focal sampling. All 16 experimental cages were assessed sequentially, resulting in a total daily observation period of approximately 160 min. No batching across different time blocks was performed; instead, all cages were observed within a single continuous session to maintain consistency in circadian timing. All cages remained in their home positions during scoring, and no handling or disturbance occurred for cages waiting to be observed, minimizing potential carry-over effects. On biweekly cage renewal days, behavioral observations were strictly performed prior to any handling procedures to avoid stress-induced behavioral artifacts. Following the 10 min behavioral assessment, subsequent measurements (body surface temperature, body weight, and food intake) were collected, after which mice were transferred to clean cages and the soiled cages were photographed for soiling analysis.

Because the observation period occurred during the light phase, when mice are typically less active, a brief, standardized stimulus (e.g., light tapping or slight movement of the cage) was introduced immediately prior to the start of the observation. This gentle disturbance was applied consistently across all cages to promote wakefulness and capture active behavior under comparable conditions while minimizing stress. During the assessment, cages remained to their home positions with the lid closed. Observations were conducted by two educated personnel employed by the laboratory animal care unit, both of whom were highly familiar with behavioral assessment procedures. Both observers scored the behaviors simultaneously but independently. At the end of the process, their measurements were compared, and the final numerical value for the cage was determined by the median of the two observers’ scores to mitigate individual bias. While a formal inter-observer reliability coefficient was not calculated—which we acknowledge as a methodological limitation—these combined measures ensured a high degree of observational consistency.

All examined behaviors were quantified on an ordinal scale of 1–3 (1: no or minimal expression, 2: moderate expression, 3: great expression) and subsequently averaged per experimental group (Table 1). A behavioral bout was defined as a continuous episode separated from subsequent occurrences by at least 2–3 s of inactivity or a clear transition to a different behavior, in accordance with established ethological methodology [34]. Very brief or ambiguous movements (<3–5 s) were not considered valid behavioral bouts in order to reduce noise from transient activity. Behavioral scoring differed between categories depending on the ethological nature of each behavior. State-like behaviors (e.g., locomotion, resting, nest building) were quantified primarily by duration, as these behaviors are continuous and best represented by time allocation within the observation window [35,36]. In contrast, event-like behaviors (e.g., feeding, drinking, agonistic interactions, and discrete digging bouts) were quantified by frequency, as these behaviors occur in distinct episodes rather than sustained states. Although grooming is considered a state-like behavior, it was quantified here using bout frequency categories for standardized behavioral scoring. This dual approach allows for a more accurate representation of behavioral structure while maintaining comparability across experimental groups.

### 2.7. Statistical Analysis

Statistical analyses were performed using R v.4.2.3 and the packages tidyverse [37], car [38], emmeans [39], rstatix [40], broom [41], ggdist [42], ggpubr [40], and ggsci [43]. Prior to inferential testing, data distributions were evaluated for normality using the Shapiro–Wilk test, and homogeneity of variances was confirmed using Levene’s test to validate the assumptions required for parametric statistics. Physiological parameters—namely body surface temperature (BST) and food intake (FI)—were analyzed using a three-way analysis of variance (ANOVA) with Type II sums of squares [44]. To accurately assess the effect of the housing environment on growth while controlling for initial size variations, body weight (BW) profiles were analyzed using a robust Analysis of Covariance (ANCOVA) with the baseline body weight (Day 1) set as a continuous covariate. Intra-cage environmental factors, such as cage temperature (CT) and cage humidity (CH), were analyzed via unpaired two-tailed Student’s *t*-tests [45] assuming equal variance. Following ANOVA and ANCOVA models, main effects and interactions of genotype (129SV vs. Des−/−), sex (Male ♂ vs. Female ♀), and bedding condition (NB vs. DB) were also evaluated. Significant effects were calculated by post hoc pairwise comparisons using estimated marginal means adjusted with Tukey’s honest significant difference (HSD) test [46]. Data distributions were visualized as raincloud plots. Furthermore, relationships between external environmental factors and the microenvironment of each experimental cage unit, namely “Room Temperature (RT) vs. Cage Temperature (CT)” and “Room Humidity (RH) vs. Cage Humidity (CH)”, were assessed using linear regression models stratified by bedding condition [47]. For linear regression models, the calculated *p*-values assess whether the slope of the fitted line is significantly different from zero, indicating a predictive relationship between the room and cage environments. To maintain statistical rigor and account for the multifactorial design, the level of bedding surface soiling was analyzed via three-way ANOVA. To strictly avoid pseudoreplication, all sequentially measured values taken from the same cage were aggregated to a single mean value per cage prior to analysis, preserving the cage as the true experimental unit. Lastly, intra-cage behavioral repertoires were quantified as frequency scores. Statistical significance for all tests was defined as *p* < 0.05.

## 3. Results

In order to comprehensively evaluate the effectiveness of deep bedding as a method of environmental enrichment, we conducted a comprehensive evaluation across a range of physiological, environmental, and behavioral parameters in 129SV (wild-type) and Des−/− (desmin-knockout) mice housed under normal (0.4 L or 200 g) versus deep (1.6 L or 800 g) bedding volumes. Distinct, experimentally naive animals were utilized for each cohort to ensure strict consistency in starting age and baseline body weight. This design resulted in a cumulative sample size of *N* = 16 cages (64 mice in total), which served as the experimental units. In essence, the increased bedding volume significantly influenced several aspects of the cage microenvironment, including temperature and humidity, with notable genotype- and sex-specific responses. While body surface temperature remained largely unaffected, body weight, food intake, bedding surface soiling, and behavioral expression were differentially modulated by bedding depth. Collectively, our findings highlight the multifactorial influence of bedding volume on both environmental conditions and animal physiology as detailed in the following subsections.

### 3.1. Modulation of the Room-to-Cage Thermal Gradient by Bedding Volume

**Figure 2:**
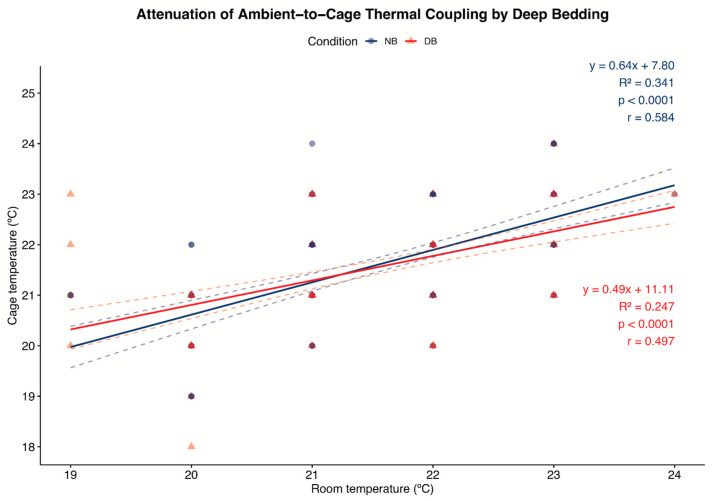
Linear regression analysis illustrating the relationship between ambient room temperature (*x*-axis) and intra-cage microclimate temperature (*y*-axis) stratified by bedding condition. Data points represent individual measurements for Normal Bedding (NB, blue circles) and Deep Bedding (DB, red triangles). Solid lines indicate the best-fit linear regression models, while dashed lines represent the 95% confidence intervals for each group. Statistical annotations display the regression equation (y = mx + c), coefficient of determination (R^2^), *p*-value, and Pearson correlation coefficient (r) for each condition.

Comparison of the mean cage temperature values between the NB and DB groups did not yield any statistical significance (*p* = 0.479), averaging at 21.74 ± 0.09 °C and 21.66 ± 0.08 °C, respectively (Supplementary Table S1). Linear regression and Pearson correlation analyses revealed that deep bedding significantly alters the thermodynamic coupling between the cage microenvironment and ambient room conditions (Figure 2). While intra-cage temperature remained positively correlated with room temperature across both groups (with both individual regression slopes significantly different from zero, *p* < 0.0001), the numerical strength of this association was attenuated under deep bedding (DB). Normal bedding (NB) cages exhibited a robust linear coupling to ambient fluctuations (r = 0.584, 95% CI [0.47, 0.68]), characterized by a steep response slope (0.64 ± 0.07) and higher explanatory power (R^2^ = 0.341). In contrast, DB cages displayed a numerically weaker correlation (r = 0.497, 95% CI [0.37, 0.61]) and a shallower response slope (0.49 ± 0.07; R^2^ = 0.247), alongside a higher thermal intercept (11.1 °C vs. 7.8 °C). While these metrics mathematically demonstrate that increased bedding volume provides a measurable thermal buffering effect against external temperature variability, the overlapping confidence intervals and the strictly controlled ambient room temperature (21 ± 2 °C) limit the absolute magnitude of this insulation in practice. Given these realistic facility values, the thermal decoupling observed here serves primarily as a physical proof-of-concept of the substrate’s properties rather than a driver of severe microclimatic alteration.

### 3.2. Modulation of the Room-to-Cage Hygrometric Gradient by Bedding Volume

**Figure 3:**
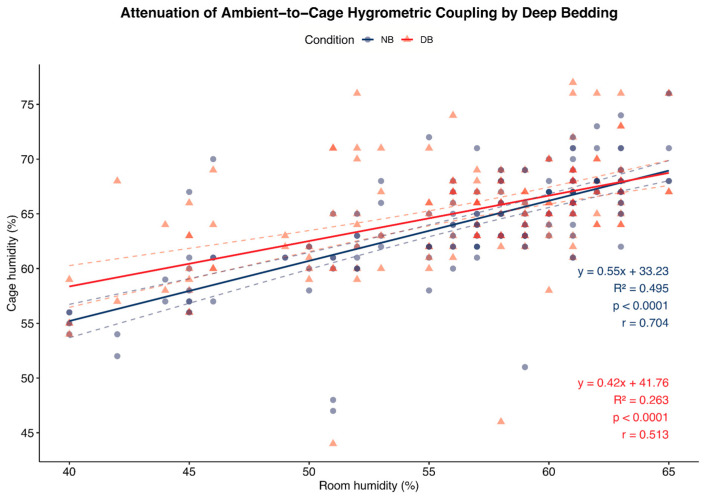
Linear regression analysis illustrating the relationship between ambient room humidity (*x*-axis) and intra-cage humidity (*y*-axis) stratified by bedding condition. Data points represent individual measurements for Normal Bedding (NB, blue circles) and Deep Bedding (DB, red triangles). Solid lines indicate the best-fit linear regression models, while dashed lines represent the 95% confidence intervals for each group. Statistical annotations display the regression equation (y = mx + c), coefficient of determination (R^2^), *p*-value, and Pearson correlation coefficient (r) for each condition.

Comparison of the mean cage humidity values between the NB and DB groups did not yield any statistical significance (*p* = 0.079), averaging at 64.21 ± 0.38% and 65.16 ± 0.39%, respectively (Supplementary Table S2). Linear regression and Pearson correlation analyses demonstrated that deep bedding significantly attenuates the influence of ambient humidity on the cage microenvironment (Figure 3). While intra-cage humidity tracked room conditions in both groups (with both individual regression slopes significantly different from zero, *p* < 0.0001), the magnitude of this coupling was numerically reduced under deep bedding (DB). Standard housing (NB cages) exhibited a robust linear response to ambient fluctuations (r = 0.704, 95% CI [0.62, 0.77]; slope = 0.55 ± 0.04), with room humidity explaining approximately 50% of the total variance in cage microclimate (R^2^ = 0.495). In contrast, DB cages displayed a numerically weaker correlation (r = 0.513, 95% CI [0.39, 0.62]; R^2^ = 0.263) and a shallower response slope (0.42 ± 0.06), alongside a higher hygrometric baseline/intercept (41.8% vs. 33.2%). While these data indicate that increased bedding volume provides a measurable hygrometric buffering effect, the overlapping confidence intervals and the tightly regulated facility humidity (55% ± 10%) suggest that the practical biological impact of this decoupling is minimal. As with temperature, this relationship acts as a physical demonstration of the substrate’s moisture-retentive properties rather than a severe environmental shift.

### 3.3. Effect of Deep Bedding on Mouse Body Surface Temperature

Assessment of mouse body surface temperature (BST) revealed a remarkable physiological stability across all experimental cohorts (Table 2, Supplementary Tables S3 and S4). A three-way ANOVA demonstrated that surface thermoregulation was unaffected by genotype (F_1,34_ = 0.366, *p* = 0.549), sex (F_1,34_ = 1.32, *p* = 0.259), or bedding volume (F_1,34_ = 0.618, *p* = 0.437). Furthermore, no significant interactions were detected between these factors, indicating that the thermoregulatory profile remains consistent regardless of genetic background or environmental enrichment status. Descriptive analysis confirmed this uniformity, with the mean and median BST values restricted to a narrow physiological window across the groups, as detailed in Table 2. Although differences in BST greater than 0.5 °C are typically considered biologically significant, the absence of statistical support for any pairwise contrast—coupled with high residual variance (88.6%)—indicates that the observed fluctuations reflect intrinsic individual variability rather than a functional deviation in thermal phenotype.

### 3.4. Effect of Deep Bedding on Mouse Body Weight

**Figure 4:**
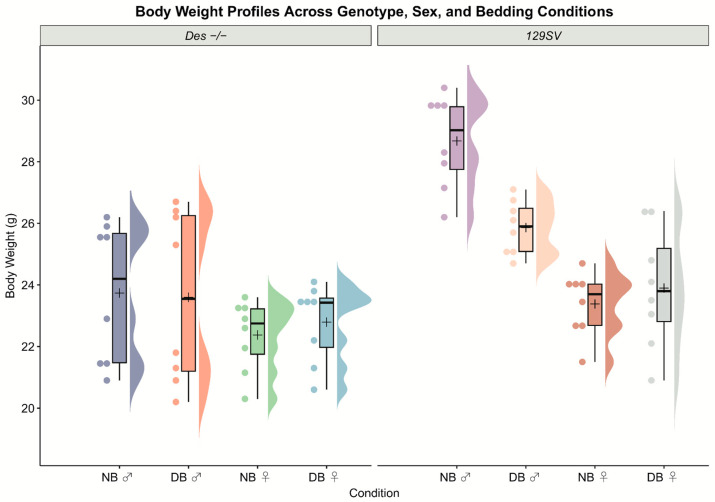
Raincloud plots illustrating the distribution of body weight (g) in Des−/− ((**left**) panel) and 129SV ((**right**) panel) mice. Data are stratified by sex (Male: ♂, Female: ♀) and bedding volume (NB: Normal Bedding; DB: Deep Bedding). Each data cluster represents the distribution for a specific experimental group and includes: (1) individual data points (“rain”) showing raw variability, (2) a box-and-whisker plot indicating the median values (solid center line), mean values (cross, “+”), interquartile range (IQR; box boundaries), and 1.5 × IQR (whiskers), and (3) a half-violin density plot (“cloud”) visualizing the probability distribution shape.

To accurately assess the effect of the housing environment on growth while controlling for initial size variations, body weight profiles were analyzed using a robust Analysis of Covariance (ANCOVA) with the baseline body weight (Day 1) set as a continuous covariate (Figure 4, Supplementary Tables S5–S8). The ANCOVA revealed that subsequent body weight was highly dependent on the initial baseline weight (F_1,55_ = 135.31, *p* < 0.0001) and genetic background, with the 129SV strain exhibiting naturally higher mass than the Des−/− cohort (F_1,55_ = 13.79, *p* < 0.001). Crucially, after adjusting for baseline weight, neither bedding volume (*p* = 0.54) nor sex (*p* = 0.23) significantly modulated body weight, and all higher-order interactions were strictly non-significant (all *p* > 0.50). Robust post hoc comparisons holding the covariate constant confirmed that there were no significant weight differences between normal and deep bedding within any specific genotype or sex cohort (all adjusted *p* > 0.93). These covariate-adjusted results demonstrate that deep bedding does not physiologically alter growth trajectories or mass maintenance; any isolated group differences observed prior to adjustment were strictly the result of baseline randomization artifacts rather than a true response to the enriched environment.

### 3.5. Effect of Deep Bedding on Food Intake

Analysis of food intake identified genetic background as the predominant driver of variability in this trait (Table 3, Supplementary Tables S9–S11). A three-way ANOVA revealed a significant main effect of Genotype (F_1,49_ = 4.52, *p* = 0.039), with Des−/− mice exhibiting elevated baseline levels relative to the 129SV strain. A marginal trend toward sexual dimorphism was also observed (F_1,49_ = 3.84, *p* = 0.056), suggesting a propensity for higher values in females, though this did not reach the threshold for statistical significance. Notably, this genetic influence persisted independently of environmental context; neither bedding volume (*p* = 0.60) nor any higher-order interactions (*p* > 0.28) significantly modulated the outcome. While the overall effect of genotype was statistically significant, high within-group heterogeneity—evidenced by substantial residual variance (82.3%)—precluded the detection of specific pairwise differences in post hoc comparisons (all *p* > 0.46). Collectively, these data characterize food intake as a trait governed primarily by intrinsic genetic and individual variability, remaining resilient to modification by environmental bedding enrichment.

### 3.6. Effect of Deep Bedding on Cage Bedding Soiling

**Figure 5:**
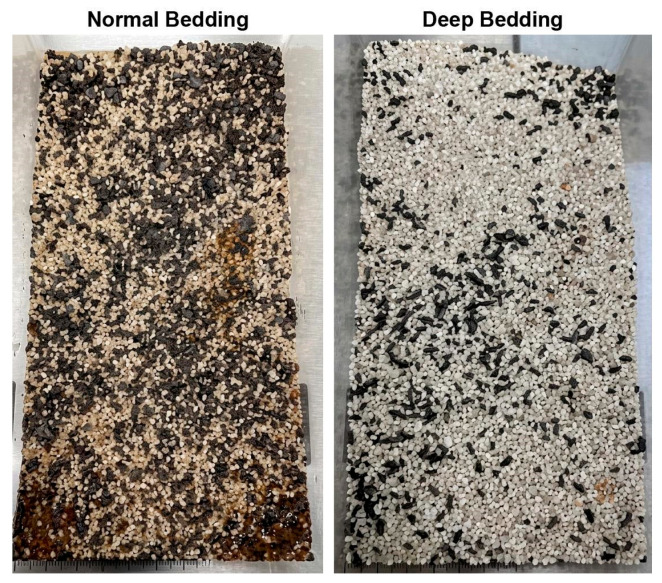
Representative images showing the levels of bedding soiling in groups of normal (low) and deep (high) bedding volume. All pictures were taken after the bi-weekly cage change (14 days after housing the mice to the specific cages). The left-side image corresponds to groups of mice housed in normal bedding, where the extend of soiled surface is significantly higher in comparison to the right-side image, taken from groups of mice housed in deep bedding.

**Figure 6:**
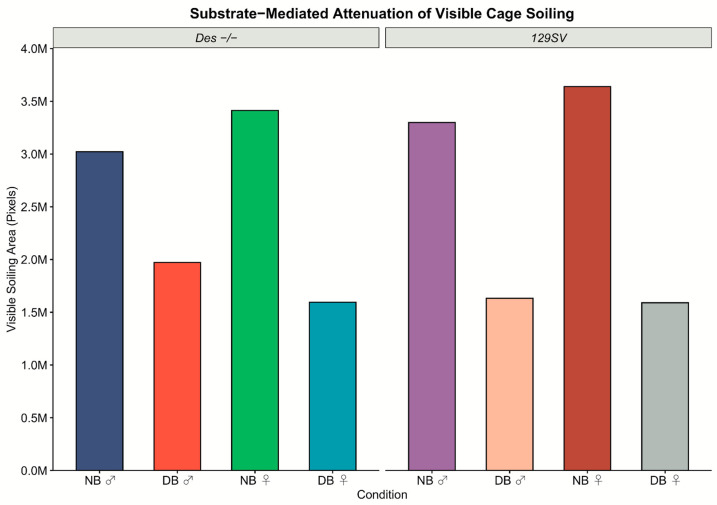
Quantification of bedding surface soiling levels (fecal surface area in pixels) stratified by genotype (Des−/−, (**left**) panel; 129SV, (**right**) panel), sex and bedding condition. A three-way ANOVA revealed a significant main effect of bedding volume (*p* < 0.01), indicating robust attenuation of soiling in DB cages across all genotypes and sexes.

Quantification of fecal surface area via Image Pro software demonstrated that increased bedding volume significantly attenuates visible cage soiling (Figure 5). A three-way ANOVA revealed a highly significant main effect of Bedding Condition (F_1,4_ = 38.03, *p* = 0.0035) (Figure 6, Supplementary Table S12). Analysis of the estimated marginal means (which average across sex and genotype to isolate the bedding effect) confirmed a massive reduction in visible soiling, dropping from a mean of 3,343,031 pixels in Normal Bedding (NB) cages to just 1,697,574 pixels in Deep Bedding (DB) cages. Importantly, the ANOVA confirmed that surface soiling was unaffected by Genotype (*p* = 0.888) or Sex (*p* = 0.784), nor were there any significant interaction effects. This indicates that the hygienic improvement provided by deep bedding is robust and uniform across all tested cohorts. While NB cages appeared heavily soiled by the end of the biweekly cycle, daily observations confirmed that baseline welfare standards were maintained. Therefore, the stark visual contrast between groups is likely attributable to the physical properties of the enriched environment, specifically enhanced mechanical sequestration (burying) and increased absorptive capacity, which effectively mask surface waste without necessarily altering total fecal output.

### 3.7. Effect of Deep Bedding on Intra-Cage Mouse Behavior

**Figure 7:**
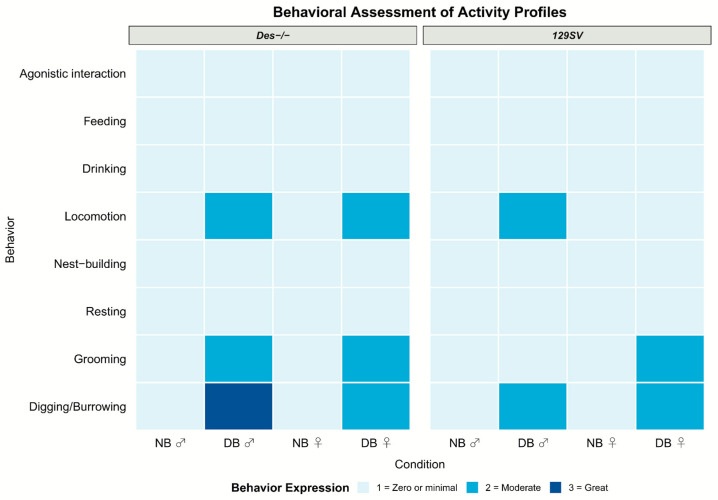
Heatmap comparing the qualitative expression of eight distinct behaviors in Des−/− and 129SV subjects. Expression levels are categorized across male (♂) and female (♀) subjects under two experimental conditions (NB and DB). The color scale indicates behavioral intensity: light blue = zero or minimal (assigned score 1), medium blue = moderate (assigned score 2), and dark blue = great (assigned score 3).

Intra-cage behavioral assessments aimed at evaluating the impact of substrate depth on welfare revealed a consistent trend towards enhanced activity (Figure 7). Given the semi-quantitative nature of the ordinal scoring system and the inherent inability to blind observers due to both the obvious physical differences in bedding depth and the visible phenotypic differences in the mice, these data must be interpreted with appropriate caution as qualitative trends rather than definitive absolute quantifications. Analysis of the behavioral repertoire, visualized as a heatmap of median ordinal scores, demonstrated a uniform upregulation of fossorial activity: median scores for digging and burrowing shifted from a baseline of 1.0 (zero/minimal) under normal bedding to 2.0–3.0 (moderate/great) under deep bedding across all genotype and sex cohorts. While locomotion and grooming also exhibited modest, context-specific elevations in DB mice, the enhancement of these manipulative behaviors indicates that increased bedding volume may release a previously constrained behavioral drive. Collectively, while cautious interpretation is warranted, these findings suggest deep bedding acts as a biologically meaningful enrichment strategy that facilitates the expression of essential naturalistic behaviors.

## 4. Discussion

The primary objective of this study was to investigate the effect of deep bedding in the cage microenvironment of 129SV (wild-type) and desmin-knockout (Des−/−) mice. In summary, our findings demonstrate that deep bedding acts as a significant environmental buffer, effectively attenuating the thermal and hygrometric coupling between the ambient room and the intra-cage environment. Crucially, this modification did not significantly alter core physiological baselines; body surface temperature (*p* = 0.437), body weight (*p* = 0.54), and food intake (*p* = 0.60) remained statistically unaffected across all cohorts. Conversely, deep bedding yielded a highly significant reduction in visible cage surface soiling (*p* = 0.0035) and facilitated a robust upregulation of natural fossorial behaviors (digging and burrowing). The Des−/− model was specifically selected due to its widespread use in cardiovascular research and its phenotype of cardiomyopathy and exercise intolerance. Given that even modest environmental stressors can alter cardiovascular parameters, the experimental design aimed to create a stable microenvironment without introducing the high-intensity physical or sensory stimulation associated with complex enrichment (e.g., running wheels or toys). By contrasting the deep bedding intervention with standard husbandry across a range of physiological, environmental, and behavioral parameters, we aimed to validate a “welfare-supportive, low-impact” strategy that does not confound the physiological baseline of fragile strains.

Historically, the drive for standardization in biomedical research and animal experimental models has favored reductionism, aiming to eliminate environmental variables to reduce noise and increase test precision. However, as André et al. [14] and Kentner et al. [11] argue, this approach often creates a “deprived” phenotype characterized by abnormal neurobiology and suppressed ethological repertoire. Among the microenvironmental variables, which significantly influence both mouse physiology and experimental outcomes, is bedding volume. Studies on bedding volume in laboratory mouse housing have revealed consistent benefits from deeper bedding in the past. Freymann et al. [48] found that increasing bedding volume per type III cage reduced corticosterone levels and adrenal, liver, kidney, and heart weights while increasing tail lengths in BALB/c and C57BL/6 mice, indicating alleviation of cold stress without raising experimental variation. Group-housed male mice preferred larger volumes, and deeper bedding supported burrowing for thermoregulation, mimicking warmer conditions. Rosenbaum et al. [49] also showed that higher bedding volumes improved microenvironmental conditions and mouse health over extended cage-change intervals in ventilated cages.

Building upon this concept, our findings further support a shift in animal lab welfare by demonstrating that deep bedding acts as a microenvironmental buffer and promoter of natural behaviors rather than a variable generator. Notably, the statistically significant attenuation of thermal and hygrometric coupling between the room and the cage (Figure 2 and Figure 3) physically insulates the subject from facility-level fluctuations. While the absolute magnitude of this buffering is modest within realistic, climate-controlled facility ranges, it aligns with Bayne [4], who posits that refinement should aim to stabilize the animal’s interaction with its environment. Nevertheless, deep bedding conditions were not able to alter overall mean cage temperature values as a result of better thermal insulation. Subsequently, no statistically or biologically significant difference was observed regarding the body surface temperature of mice housed in different conditions (Table 2), which is not in agreement with Freymann’s findings. However, Rosenbaum et al. [49], who used similar amounts of bedding material for the experimental design as in this study, also concluded that temperature values showed no significant difference between cages of different bedding volumes, suggesting that absolute cage temperature is mainly influenced by ambient temperatures.

Another critical concern regarding deep bedding has been the potential for altered metabolic rates to confound longitudinal studies. Freymann et al. [48] and Gordon [18] have previously reported that increased nesting material can elevate body surface temperature and reduce food intake, presumably by lowering the thermogenic set-point. Our results refine this understanding by introducing the concept of “Bioenergetic Reallocation”. While we observed the expected thermal buffering, we did not observe a significant reduction in food intake (Table 3). Instead, we documented a significant upregulation in fossorial activity (Figure 7). This suggests a potential homeostatic trade-off: energy conserved from reduced thermoregulatory demands may be reinvested into high-cost species-specific behaviors such as burrowing, rather than simply being stored (which would increase weight) or ignored (which would reduce intake). It should be noted, however, that this interpretation remains speculative, as thermogenic mechanisms such as shivering or non-shivering thermogenesis were not directly assessed in the present study. Therefore, this proposed explanation serves as a hypothesis warranting further investigation. If confirmed, it suggests that deep bedding does not induce a ‘sedentary, warm’ phenotype, but rather an ‘active, thermoneutral’ phenotype, which is arguably a more physiologically relevant baseline for translational modeling.

The successful adaptation of the Des−/− mice to deep bedding is a finding of significant translational relevance. The Des−/− genotype is characterized by profound mitochondrial mislocalization and respiratory defects, rendering the myocardium hypersensitive to metabolic demand [23,50]. In standard housing (20–22 °C), which is significantly below the murine thermoneutral zone (30 °C), the requirement to maintain core temperature via non-shivering thermogenesis imposes an obligate increase in heart rate and cardiac output [24]. For a cardiomyopathy model, this constitutes a continuous, unmonitored cardiac stress test. Our data suggests that deep bedding may function as a “therapeutic environment” for such fragile models. By enhancing thermal insulation and reducing the thermoregulatory burden, deep bedding theoretically lowers the basal hemodynamic load on the Des−/− heart [27]. However, because heart rate and other direct cardiovascular parameters were not measured in the current study, any interpretation regarding mitigated cardiac workload remains speculative and warrants confirmation through targeted in vivo physiological monitoring. The absence of adverse events in the deep bedding group, despite the increased physical exertion of digging, implies that the voluntary nature of this species-typical behavior is non-deleterious. This aligns with the biological perspective that reducing environmental stressors can mask or mitigate the penetrance of cytoskeletal defects [51]. Consequently, deep bedding improves the welfare of these animals not merely by providing comfort, but by actively reducing the allostatic load on their compromised physiology.

Finally, the “mechanical sequestration” of waste observed in this study offers a mechanistic explanation for improved hygiene scores. From image analysis, there was significantly lower soiling in deep bedding cages compared to normal bedding conditions (Figure 5 and Figure 6). While the increased volume of bedding undoubtedly contributes to the dilution of waste, its primary benefit extends significantly beyond simple dilution. The extra bedding provides a three-dimensional substrate that allows mice to actively reduce their direct and continuous contact with accumulated waste. By allowing mice to segregate their living space vertically—effectively separating resting and nesting areas from elimination zones—deep bedding restores a fundamental ethological capability as described by Makowska et al. [52]. While mice exhibit a strong preference for spatial segregation of nesting and elimination areas, standard housing conditions can constrain this behavior, leading to the accumulation of waste within resting zones [53]. The implementation of deep bedding facilitates this separation, resulting in a model organism that possesses greater agency and improved hygiene, factors that contribute to high-quality, reproducible data.

While benefits of environmental enrichment practices, such as nesting material, are well defined, the present study focused on isolating the effects of bedding depth under standardized housing conditions. Corncob bedding, although sometimes considered less optimal for nesting than paper-based materials, is widely used in laboratory mouse housing and provides a consistent baseline substrate. In contrast to nesting material, which is not uniformly provided across facilities and may introduce variability in use and maintenance, bedding represents a standard and continuously present component of the home cage environment. Accordingly, bedding depth constitutes a more practical and standardized parameter for experimental manipulation. Previous guidelines and studies have identified bedding as a core husbandry element with direct effects on welfare and behavior [54].

Nevertheless, it is important to note a limitation regarding the behavioral assessment in this study. The ethogram utilized here was designed to capture broad behavioral shifts in response to bedding volume rather than to provide a comprehensive affective welfare assessment. Consequently, abnormal repetitive behaviors (e.g., stereotypies) and the ‘inactive but awake’ (IBA) state were not explicitly differentiated from general resting. We acknowledge that IBA has recently been identified as a sensitive indicator of negative affect and depression-like states in laboratory mice [55]. The explicit inclusion and quantification of these specific welfare-associated behaviors would significantly strengthen future investigations into the psychological impacts of deep bedding. Additionally, to prevent baseline physiological variations from introducing randomization artifacts into longitudinal data—such as the baseline body weight discrepancies observed in our initial raw cohorts—future environmental enrichment studies should avoid simple randomization. Instead, researchers are advised to employ stratified randomized block designs utilizing automated allocation tools (e.g., RandoMice [56]) to ensure perfect equilibration of starting weights and ages across all experimental groups.

From a practical and husbandry perspective, increasing bedding depth may offer several operational advantages in addition to its potential behavioral benefits. While deeper bedding may increase the quantity of material used per cage, it may also extend cage-change intervals by improving absorption capacity and overall cage hygiene, thereby potentially reducing both labor demands and handling frequency. Less frequent cage changing is likely to minimize disturbance to the animals and reduce handling-associated stress. In addition, a reduction in cage-change frequency may decrease staff exposure to allergens and fecal by-products. Although cost-effectiveness was not directly evaluated in the present study, the potential trade-off between increased material use and reductions in labor, animal disturbance, and staff exposure represents an important consideration that warrants further investigation.

## 5. Conclusions

In conclusion, we establish deep bedding as a multifactorial refinement strategy that harmonizes ethological welfare with robust experimental reproducibility. By functionally decoupling the intra-cage microclimate from ambient fluctuations, this intervention creates a stable thermal niche that minimizes environmental stress without compromising physiological integrity, even in pathologically sensitive Des−/− models. Crucially, the observed homeostatic stability in metabolic parameters suggests a beneficial bioenergetic shift, wherein energy conserved from reduced thermoregulatory demand is reallocated toward an upregulation of species-specific fossorial behaviors. Furthermore, the mechanism of mechanical sequestration improves hygiene profiles, supporting extended husbandry intervals that reduce cumulative handling stress. Collectively, these findings define deep bedding as a superior, low-variation standard of care that resolves the historic conflict between environmental enrichment and data integrity, benefiting both the biological subject and the scientific instrument.

## Figures and Tables

**Table 1:** Ethogram used for intra-cage behavior analysis.

Category	Definition	Scoring
Agonistic Behavior	Rushing and leaping a partner with kicks and bites, chasing a fleeing partner; Fleeing, moving away from attacking partner. Each interaction separated by ≥2–3 s is considered a distinct event.	1: 0 events
		2: 1–3 events
		3: ≥4 events
Feeding	Active ingestion of food (sniffing + biting/chewing while at food source). Continuous engagement defined as a feeding bout.	1: 0–1 bout
		2: 2–4 bouts
		3: ≥5 bouts
Drinking	Water consumption via the water bottle.	1: 0–1 bout
		2: 2–4 bouts
		3: ≥5 bouts
Locomotion	Active movement including walking, running, climbing, or jumping. Measured as total time engaged in movement.	1: <2 min
		2: 2–4 min
		3: >4 min
Nest Building	All behaviors linked with nest-building (arranging, pulling in, fraying of bedding material)	1: <1 min engagement
		2: 1–4 min
		3: >4 min
Resting	Immobile posture (sitting, lying, curled posture) with eyes open or closed, not engaged in other behaviors.	1: <2 min
		2: 2–4 min
		3: >4 min
Grooming	Self-directed cleaning behavior including licking, nibbling, and wiping of fur or body parts. Defined as continuous grooming bout separated by ≥2–3 s pause.	1: 0–1 bout
		2: 2–4 bouts
		3: ≥5 bouts
Digging, burrowing	Repetitive forelimb and/or hindlimb movements displacing bedding or substrate.	1: 0–1 bout
		2: 2–4 bouts
		3: ≥5 bouts

**Table 2:** Summary statistics of body surface temperature (°C) across genotype, sex, and bedding conditions.

Genotype	Sex	Bedding Condition	Mean (°C)	Median (°C)	Interquartile Range	Standard Error
Des−/−	Male (♂)	Normal Bedding	35.8	35.9	0.21	0.06
		Deep Bedding	35.4	36	0.55	0.54
	Female (♀)	Normal Bedding	35.9	35.9	0.26	0.08
		Deep Bedding	35.9	35.9	0.3	0.07
129SV	Male (♂)	Normal Bedding	36	36	0.15	0.09
	Male (♂)	Deep Bedding	35.9	35.9	0.15	0.09
	Female (♀)	Normal Bedding	35.8	35.8	0.1	0.06
	Female (♀)	Deep Bedding	36	35.9	0.1	0.07

**Table 3:** Summary statistics of food intake (g) across genotype, sex, and bedding conditions.

Genotype	Sex	Bedding Condition	Mean (g)	Median (g)	Standard Error	Interquartile Range
Des−/−	Male (♂)	Normal Bedding	136.37	147.2	49.3	13.21
		Deep Bedding	156.93	156.3	51.95	13.08
	Female (♀)	Normal Bedding	178.26	146.8	94.5	20.4
		Deep Bedding	171.31	162.4	63.6	15.59
129SV	Male (♂)	Normal Bedding	127.43	116.8	37.2	9.4
		Deep Bedding	138.87	129.6	24.6	6.79
	Female (♀)	Normal Bedding	147.23	131.4	16.8	15.79
		Deep Bedding	142.77	129.4	39.5	16.31

## Data Availability

The data that support the findings of this study are available within the article and its Supplementary Material Files. No additional external data archives were used.

## Supplementary Materials

The following supporting information can be downloaded at: https://www.mdpi.com/article/10.3390/ani16111585/s1, Table S1: Summary statistics for the linear regression analyses modeling the relationship between ambient room temperature and intra-cage microclimate temperature, stratified by Normal Bedding and Deep Bedding conditions; Table S2: Summary statistics for the linear regression analyses modeling the relationship between ambient room humidity and intra-cage microclimate humidity, stratified by Normal Bedding and Deep Bedding conditions; Table S3: Evaluations for normality of residuals and homogeneity of variance; Table S4: Analysis of Variance (Type II) evaluating the main effects and interactions of Genotype, Sex, and Bedding on body surface temperature; Table S5: Summary statistics of body weight (g) across genotype, sex, and bedding conditions; Table S6: Evaluations for normality of residuals and homogeneity of variance; Table S7: Three-way Analysis of Covariance evaluating the main effects and interactions of Genotype, Sex, and Bedding on Body Weight, using Day 1 Body Weight as a continuous covariate; Table S8: Post-hoc Pairwise Comparisons (Covariate Adjusted)Tukey-adjusted pairwise comparisons holding the baseline weight covariate constant; Table S9: Evaluations for normality of residuals and homogeneity of variance; Table S10: Three-way Analysis of Variance evaluating the main effects and interactions of Genotype, Sex, and Bedding on Food Intake; Table S11: Post-hoc Tukey-adjusted pairwise comparisons; Table S12: Analysis of Variance for Bedding Surface Soiling.
