# Supplementary material for: Impact of Bedding Volume on Cage Microclimate and Behavior in 129SV and Desmin-Knockout (Des−/−) Mice
Source: Animals (Basel). 2026 May 23;16(11):1585. doi: 10.3390/ani16111585 (PMC13255793; doi:10.3390/ani16111585)
Supplement: Supplementary file 1 [file animals-16-01585-s001.zip › animals-4265245-supplementary.pdf]

Supplementary Table S1. Summary statistics for the linear regression analyses modeling the relationship between ambient room temperature and intra-cage microclimate temperature, stratified by Normal Bedding and Deep Bedding conditions

Bedding Condition	Intercept (°C)	Slope	R^2	Pearson r	95% CI for r	p-value (slope = 0)
Normal Bedding	7.8	0.641	0.341	0.584	[0.471, 0.678]	5.33×10^{-16}
Deep Bedding	11.1	0.485	0.247	0.497	[0.370, 0.605]	2.37×10^{-11}

Supplementary Table S2. Summary statistics for the linear regression analyses modeling the relationship between ambient room humidity and intra-cage microclimate humidity, stratified by Normal Bedding and Deep Bedding conditions

Bedding Condition	Intercept (%)	Slope	R^2	Pearson r	95% CI for r	p-value (slope = 0)
Normal Bedding	33.2	0.549	0.495	0.704	[0.62, 0.77]	3.29×10^{-25}
Deep Bedding	41.8	0.415	0.263	0.513	[0.39, 0.62]	4.03×10^{-12}

Supplementary Table S3. Evaluations for normality of residuals and homogeneity of variance.

Test	Null Hypothesis (H0)	Statistic	p-value	Conclusion
Shapiro-Wilk	Residuals are normally distributed	W = 0.560	< 0.001 ***	Normality violated a
Levene's Test	Variances are equal across groups	F(7, 34) = 1.014	0.439 ns	Equal variance assumed

Supplementary Table S4. Analysis of Variance (Type II) evaluating the main effects and interactions of Genotype, Sex, and Bedding on body surface temperature.

Source of Variation	Degrees of Freedom	Sum of Squares (SS)	F-value	p-value	% Total Variation	Significance

	(df)					
Genotype	1	0.141	0.366	0.549	0.95%	ns
Sex	1	0.507	1.32	0.259	3.44%	ns
Bedding	1	0.237	0.618	0.437	1.61%	ns
Genotype × Sex	1	0.27	0.703	0.408	1.83%	ns
Genotype × Bedding	1	0.142	0.37	0.547	0.96%	ns
Sex × Bedding	1	0.371	0.965	0.333	2.51%	ns
Genotype × Sex × Bedding	1	0.013	0.033	0.857	0.09%	ns
Residuals	34	13.06	-	-	88.60%	-

Supplementary Table S5. Summary statistics of body weight (g) across genotype, sex, and bedding conditions.

Genotype	Sex	Bedding Condition	Mean (g)	Median (g)	Interquartile Range	Standard Error
Des -/-	Male (♂)	Normal Bedding	23.70	24.20	4.20	0.81
		Deep Bedding	23.60	23.60	5.05	0.99
	Female (♀)	Normal Bedding	22.40	22.80	1.47	0.41
		Deep Bedding	22.80	23.40	1.60	0.45
129SV	Male (♂)	Normal Bedding	28.70	29.00	2.04	0.53
		Deep Bedding	25.90	25.90	1.40	0.31
	Female (♀)	Normal Bedding	23.40	23.70	1.34	0.37
		Deep Bedding	23.90	23.80	2.38	0.68

Supplementary Table S6. Evaluations for normality of residuals and homogeneity of variance.

Assumption Test	Statistic	p-value	Interpretation

Shapiro-Wilk Test (Normality of Residuals)	W = 0.974	0.19	Residuals are normally distributed.
Levene's Test (Homogeneity of Variance)	F(7, 56) = 5.628	< 0.001 ***	Assumption of equal variances is violated; justifies the use of White-adjusted robust ANCOVA.

Supplementary Table S7. Three-way Analysis of Covariance evaluating the main effects and interactions of Genotype, Sex, and Bedding on Body Weight, using Day 1 Body Weight as a continuous covariate.

Source of Variation	Degrees of Freedom (df)	F-value	p-value	Significance
Baseline Weight (Covariate)	1, 55	135.31	< 0.0001	****
Genotype	1, 55	13.79	0.00048	***
Sex	1, 55	1.43	0.236	ns
Bedding	1, 55	0.37	0.545	ns
Genotype × Sex	1, 55	0.13	0.719	ns
Genotype × Bedding	1, 55	0.46	0.501	ns
Sex × Bedding	1, 55	0.03	0.874	ns
Genotype × Sex × Bedding	1, 55	0.07	0.794	ns
Residuals	55	-	-	-

*(Note: ns = not significant, *** p < 0.001, **** p < 0.0001)*

Supplementary Table S8. Post-hoc Pairwise Comparisons (Covariate Adjusted)Tukey-adjusted pairwise comparisons holding the baseline weight covariate constant.

Comparison (Group 1 vs Group 2)	Estimate (Δ)	SE	df	t-ratio	Adjusted p-value
Des -/- NB ♂ vs. Des -/- DB ♂	-0.288	0.424	55	-0.679	> 0.99 (ns)

Des -/- NB ♂ vs. Des -/- NB ♀	0.271	0.439	55	0.618	> 0.99 (ns)
Des -/- NB ♂ vs. Des -/- DB ♀	0.001	0.437	55	0.001	1 (ns)
Des -/- DB ♂ vs. Des -/- NB ♀	0.559	0.47	55	1.189	0.932 (ns)
Des -/- DB ♂ vs. Des -/- DB ♀	0.288	0.475	55	0.607	> 0.99 (ns)
Des -/- NB ♀ vs. Des -/- DB ♀	-0.271	0.486	55	-0.557	> 0.99 (ns)
129SV NB ♂ vs. 129SV DB ♂	0.168	0.558	55	0.301	> 0.99 (ns)
129SV NB ♂ vs. 129SV NB ♀	0.597	0.668	55	0.894	0.985 (ns)
129SV NB ♂ vs. 129SV DB ♀	0.522	0.676	55	0.772	0.994 (ns)
129SV DB ♂ vs. 129SV NB ♀	0.429	0.546	55	0.785	0.993 (ns)
129SV DB ♂ vs. 129SV DB ♀	0.354	0.569	55	0.621	> 0.99 (ns)
129SV NB ♀ vs. 129SV DB ♀	-0.075	0.563	55	-0.133	1 (ns)

(Note: NB = Normal Bedding, DB = Deep Bedding, ns = not significant)

Supplementary Table S9. Evaluations for normality of residuals and homogeneity of variance.

Test	Assumption Evaluated	Statistic	p-value	Conclusion
Shapiro-Wilk	Normality of Residuals	W = 0.9557	0.036	Slight deviation from normality
Levene's Test	Homogeneity of Variance	F = 0.7888	0.6001	Variances are equal (assumptions met)

Supplementary Table S10. Three-way Analysis of Variance evaluating the main effects and interactions of Genotype, Sex, and Bedding on Food Intake.

Effect	Degrees of Freedom (df)	Sum of Squares (SS)	F-value	p-value	% Total Variation	Significance
Genotype	1	6630.34	4.518	0.0386	7.59%	Yes (*)
Sex	1	5637.6	3.842	0.0557	6.45%	No (ns)
Bedding	1	415.3	0.283	0.5971	0.48%	No (ns)
Genotype : Sex	1	932.83	0.636	0.4291	1.07%	No (ns)
Genotype : Bedding	1	41.22	0.028	0.8676	0.05%	No (ns)
Sex : Bedding	1	1688.66	1.151	0.2886	1.93%	No (ns)
Genotype : Sex : Bedding	1	119.54	0.081	0.7765	0.14%	No (ns)
Residuals	49	71902.63	-	-	82.30%	-

*(Note: ns = not significant, * p < 0.05)*

Supplementary Table S11. Post-hoc Tukey-adjusted pairwise comparisons.

Comparison (Group 1 vs Group 2)	Estimate (Δ)	SE	df	t-ratio
Des -/- NB ♂ vs. Des -/- DB ♂	-20.55	19.83	-1.037	0.966 (ns)
Des -/- NB ♂ vs. Des -/- NB ♀	-41.89	20.48	-2.046	0.463 (ns)
Des -/- NB ♂ vs. Des -/- DB ♀	-34.94	20.48	-1.707	0.683 (ns)
Des -/- DB ♂ vs. Des -/- NB ♀	-21.33	19.83	-1.076	0.959 (ns)
Des -/- DB ♂ vs. Des -/- DB ♀	-14.39	19.83	-0.726	0.996 (ns)
Des -/- NB ♀ vs. Des -/- DB ♀	6.94	20.48	0.339	>0.999 (ns)
129SV NB ♂ vs. 129SV DB ♂	-11.44	20.48	-0.559	0.999 (ns)
129SV NB ♂ vs. 129SV NB ♀	-19.8	20.48	-0.967	0.977 (ns)
129SV NB ♂ vs.	-15.34	20.48	-0.749	0.995 (ns)

129SV DB ♀				
129SV DB ♂ vs. 129SV NB ♀	-8.36	20.48	-0.408	>0.999 (ns)
129SV DB ♂ vs. 129SV DB ♀	-3.9	20.48	-0.19	>0.999 (ns)
129SV NB ♀ vs. 129SV DB ♀	4.46	20.48	0.218	>0.999 (ns)

(Note: NB = Normal Bedding, DB = Deep Bedding, ns = not significant)

Supplementary Table S12. Analysis of Variance for Bedding Surface Soiling

**Effect**	**Degrees of Freedom (DF)**	**F-value**	**p-value**	**Significance**
Genotype	1	0.022	0.888	ns
Sex	1	0.085	0.785	ns
Bedding	1	38.036	0.0035	**
Residuals	4	-	-	-

*(Note: ns = not significant, ** $p < 0.01$)*
